# Supplementary material for: Polyketide synthase gene clusters in the ericoid mycorrhizal fungus Oidiodendron maius: genome-wide prediction, expression profiling, and functional characterization of knockout mutants
Source: Front Plant Sci. 2026 Sep 16;17:1924430. doi: 10.3389/fpls.2026.1924430 (PMC13623903; doi:10.3389/fpls.2026.1924430)
Supplement: Supplementary file 1 [file SupplementaryFile1.zip › Supplementary Materials/Supplementary Folder 1.zip!/Oidma1_AssemblyScaffolds_Repeatmasked/knownclusterblast/cluster39/jgi.p_Oidma1_132997_mibig_hits.html]

| MIBiG Protein | Description | MIBiG Cluster | MiBiG Product | % ID | % Coverage | BLAST Score | E-value |
| --- | --- | --- | --- | --- | --- | --- | --- |
| BAC20568.1 | efflux\_pump | BGC0000039 | Compactin\_biosynthetic\_gene\_cluster | 48 | 99.2647058824 | 498 | 9.3e-141 |
| ABA02247.1 | efflux\_pump | BGC0000098 | Monacolin\_K\_biosynthetic\_gene\_cluster | 47 | 95.5882352941 | 481 | 1.2e-135 |
| AAD34558.1 | unknown | BGC0000089 | Lovastatin\_biosynthetic\_gene\_cluster | 46 | 95.7720588235 | 467 | 1.8e-131 |
| ADY16699.1 | TqaJ | BGC0001142 | Tryptoquialanine\_biosynthetic\_gene\_cluster | 39 | 95.5882352941 | 372 | 7.7e-103 |
| CAP96439.1 |  | BGC0000420 | Roquefortine\_C\_/\_meleagrin\_biosynthetic\_gene\_cluster | 38 | 96.1397058824 | 356 | 4.4e-98 |
| AAS90069.1 | AflT | BGC0000010 | Aflatoxin\_biosynthetic\_gene\_cluster | 38 | 95.2205882353 | 349 | 9.1e-96 |
| AGN71625.1 | putative\_HC-toxin\_efflux\_carrier\_TOXA | BGC0000027 | Azaphilone\_biosynthetic\_gene\_cluster | 52 | 59.7426470588 | 344 | 2.9e-94 |
| AAS89998.1 | AflT | BGC0000007 | Aflatoxin\_biosynthetic\_gene\_cluster | 37 | 93.3823529412 | 341 | 1.5e-93 |
| AAS90046.1 | AflT | BGC0000009 | Aflatoxin\_biosynthetic\_gene\_cluster | 37 | 96.6911764706 | 340 | 3.2e-93 |
| AAS90021.1 | AflT | BGC0000008 | Aflatoxin\_biosynthetic\_gene\_cluster | 37 | 95.2205882353 | 339 | 5.5e-93 |
| BAD29973.1 | transporter\_protein | BGC0000676 | Aphidicolin\_biosynthetic\_gene\_cluster | 37 | 91.9117647059 | 339 | 7.2e-93 |
| AAS90092.1 | AflT | BGC0000006 | Aflatoxin\_biosynthetic\_gene\_cluster | 37 | 95.2205882353 | 339 | 9.4e-93 |
| XP\_002384861.1 | MFS\_multidrug\_transporter,\_putative | BGC0001234\_c2 | 4,4'-piperazine-2,5-diyldimethyl-bis-phenol\_biosynthetic\_gene | 37 | 88.0514705882 | 330 | 3.4e-90 |
| BAE71313.1 | putative\_ABC\_transporter | BGC0000004 | Aflatoxin\_biosynthetic\_gene\_cluster | 37 | 91.7279411765 | 327 | 2.2e-89 |
| EAA65599.1 | hypothetical\_protein | BGC0000022 | Asperfuranone\_biosynthetic\_gene\_cluster | 37 | 96.6911764706 | 320 | 4.5e-87 |
| AMR44276.1 | MFS\_multidrug\_transporter | BGC0001398\_c3 | Phomopsins\_biosynthetic\_gene\_cluster | 36 | 92.6470588235 | 318 | 1.3e-86 |
| CBF87265.1 | MFS\_toxin\_efflux\_pump\_(AflT),\_putative\_(AFU\_orthologue;\_AFUA\_1G12620) | BGC0000680\_c2 | Austinol/dehydroaustinol,\_locus\_1\_biosynthetic\_gene\_cluster | 38 | 87.6838235294 | 318 | 1.7e-86 |
| CCE28984.1 | probable\_DHA14-like\_major\_facilitator;\_ABC\_transporter | BGC0001365 | Clapurines\_biosynthetic\_gene\_cluster | 32 | 95.9558823529 | 301 | 2.2e-81 |
| ACD39765.1 | major\_facilitator\_superfamily\_transporter | BGC0000077 | Hypothemycin\_biosynthetic\_gene\_cluster | 34 | 94.8529411765 | 283 | 6.1e-76 |
| ACD39756.1 | major\_facilitator\_superfamily\_transporter | BGC0000076 | Hypothemycin\_biosynthetic\_gene\_cluster | 34 | 94.8529411765 | 283 | 6.1e-76 |
| AGC95323.1 | CurE | BGC0000045 | Dehydrocurvularin\_biosynthetic\_gene\_cluster | 33 | 95.4044117647 | 260 | 3.3e-69 |
| ADM34144.1 | efflux\_pump | BGC0001084 | Notoamide\_/\_stephacidin\_biosynthetic\_gene\_cluster | 32 | 93.75 | 259 | 7.3e-69 |
| AAB36607.1 | toxin\_pump | BGC0000370\_c2 | HC-toxin\_biosynthetic\_gene\_cluster | 31 | 92.4632352941 | 252 | 1.5e-66 |
| AHV78251.1 | ResE | BGC0001246 | Trans-resorcylide\_biosynthetic\_gene\_cluster | 30 | 95.5882352941 | 243 | 4.1e-64 |
| ctg1\_orf0002 |  | BGC0000688 | Copalyl\_diphosphate\_biosynthetic\_gene\_cluster | 31 | 85.1102941176 | 219 | 1.1e-56 |
| CAF60521.1 | putative\_efflux\_protein | BGC0000704 | Kanamycin\_biosynthetic\_gene\_cluster | 27 | 92.4632352941 | 190 | 7.1e-48 |
| CAF31575.1 | putative\_kanamycin\_efflux\_protein | BGC0000705 | Kanamycin\_biosynthetic\_gene\_cluster | 27 | 92.4632352941 | 190 | 7.1e-48 |
| AAF00219.1 | transporter | BGC0000276 | Urdamycin\_biosynthetic\_gene\_cluster | 27 | 84.7426470588 | 177 | 3.6e-44 |
| AFU65888.1 | DacR2 | BGC0000216 | Dactylocycline\_biosynthetic\_gene\_cluster | 27 | 88.0514705882 | 164 | 3.2e-40 |
| ADZ13560.1 | YtkR6 | BGC0000466 | Yatakemycin\_biosynthetic\_gene\_cluster | 29 | 82.7205882353 | 125 | 1.6e-28 |
| AAM77999.1 | transmembrane\_transporter | BGC0000112 | Neocarzinostatin\_biosynthetic\_gene\_cluster | 30 | 69.8529411765 | 113 | 1.1e-24 |
| ADC79648.1 | TamJ | BGC0001052 | Tirandamycin\_biosynthetic\_gene\_cluster | 27 | 75.7352941176 | 108 | 2.1e-23 |
| ABP54623.1 | drug\_resistance\_transporter,\_EmrB/QacA\_subfamily | BGC0000241 | Lomaiviticin\_biosynthetic\_gene\_cluster | 24 | 88.7867647059 | 106 | 1e-22 |
| AHZ61835.1 | Lom1 | BGC0000240 | Lomaiviticin\_biosynthetic\_gene\_cluster | 23 | 88.9705882353 | 96 | 8.2e-20 |
| BAP16698.1 | transmembrane\_transporter | BGC0000376 | JBIR-34,\_JBIR-35\_biosynthetic\_gene\_cluster | 26 | 76.8382352941 | 94 | 3.1e-19 |
| ABB05093.1 | LipEx1 | BGC0001003 | Lipomycin\_biosynthetic\_gene\_cluster | 28 | 64.7058823529 | 93 | 6.9e-19 |
| CBA11577.1 | putative\_transport\_integral\_membrain\_protein | BGC0001046 | Streptolydigin\_biosynthetic\_gene\_cluster | 25 | 82.7205882353 | 90 | 7.6e-18 |
| AGN74899.1 | EmrB/QacA\_subfamily\_drug\_resistance\_transporter | BGC0000459 | Griseoviridin\_/\_viridogrisein\_biosynthetic\_gene\_cluster | 25 | 66.7279411765 | 88 | 2.2e-17 |
| AGH68910.1 | MFS-like\_transporter | BGC0001083 | Merochlorin\_biosynthetic\_gene\_cluster | 25 | 75.9191176471 | 86 | 8.4e-17 |
